# Population structure of *Salmonella enterica* serotype Mbandaka reveals similar virulence potential irrespective of source and phylogenomic stratification

**DOI:** 10.12688/f1000research.25540.1

**Published:** 2020-09-16

**Authors:** Linto Antony, Gavin Fenske, Radhey S Kaushik, Tiruvoor G Nagaraja, Milton Thomas, Joy Scaria

**Affiliations:** 1 Department of Veterinary & Biomedical Sciences, South Dakota State University, Brookings, SD, 57007, USA; 2 South Dakota Center for Biologics Research and Commercialization, Brookings, SD, USA; 3 Department of Biology & Microbiology, South Dakota State University, Brookings, SD, 57007, USA; 4 Department of Diagnostic Medicine & Pathobiology, College of Veterinary Medicine, Kansas State University, Manhattan, KS, 66506, USA

**Keywords:** Salmonella, Mbandaka, pathogenesis, foodborne pathogen

## Abstract

**Background**: *Salmonella enterica* serotype Mbandaka ( *Salmonella* ser. Mbandaka) is a multi-host adapted Non-typhoidal *Salmonella* (NTS) that can cause foodborne illnesses in human. Outbreaks of *Salmonella* ser. Mbandaka contributed to the economic stress caused by NTS due to hospitalizations. Whole genome sequencing (WGS)-based phylogenomic analysis facilitates better understanding of the genomic features that may expedite the foodborne spread of *Salmonella* ser. Mbandaka.

**Methods**: In the present study, we define the population structure, antimicrobial resistance (AMR), and virulence profile of *Salmonella* ser. Mbandaka using WGS data of more than 400 isolates collected from different parts of the world. We validated the genotypic prediction of AMR and virulence phenotypically using an available set of representative isolates.

**Results**: Phylogenetic analysis of *Salmonella* ser. Mbandaka using Bayesian approaches revealed clustering of the population into two major groups; however, clustering of these groups and their subgroups showed no pattern based on the host or geographical origin. Instead, we found a uniform virulence gene repertoire in all isolates. Phenotypic analysis on a representative set of isolates showed a similar trend in cell invasion behavior and adaptation to a low pH environment. Both genotypic and phenotypic analysis revealed the carriage of multidrug resistance (MDR) genes in *Salmonella* ser. Mbandaka.

**Conclusions**: Overall, our results show that the presence of multidrug resistance along with adaptation to broad range of hosts and uniformity in the virulence potential, isolates of *Salmonella* ser. Mbandaka from any source could have the potential to cause foodborne outbreaks as well as AMR dissemination.

## Introduction

For the last two decades, *Salmonella* has remained the major foodborne pathogen in the U.S. according to the Centers for Disease Control and Prevention (CDC) ^1^. Non-typhoidal *Salmonella* (NTS) is estimated to cause 1 million foodborne illnesses annually ^2^. *Salmonella enterica* subspecies *enterica* serotype Mbandaka ( *Salmonella* ser. Mbandaka) has been identified by the CDC as an important outbreak-causing serotype of *Salmonella* ^3^. Classified as one of the top ten *Salmonella* serotypes that cause human foodborne illnesses in Europe, a clonal population of *Salmonella* ser. Mbandaka (sequence type ST413) has been shown to be capable of surviving for many years and associated with animal feed, poultry, and human food ^4^. Following its initial isolation from the Belgian Congo (Central Africa) in 1948, *Salmonella* ser. Mbandaka has been reported as a cause of human salmonellosis in several countries, making this serotype globally important for human and animal health ^4– 6^.

Human foodborne illnesses caused by *Salmonella* ser. Mbandaka have rarely been reported in the U.S.; nevertheless, three multistate outbreaks were reported by the CDC between 2013 and 2018 ^7– 9^. Based on annually compiled data from several sources, Hayward *et al.* reported that cattle, poultry, and pigs are the major hosts of *Salmonella* ser. Mbandaka ^10^. However, the sources of two of the abovementioned outbreaks were from food preparations ^7, 9^, indicating the spread of this multi-host adapted serotype by other means.

 Although *Salmonella* ser. Mbandaka has been involved in several multi-serotype comparative studies, its epidemiological and evolutionary characteristics are not well understood. Previous studies on *Salmonella* ser. Mbandaka have focused on either a very small number of isolates ^11^ or isolates from a specific geographical location ^4^. Comparative genomic analysis has been widely used as a powerful tool for elucidating genomic diversity across *Salmonella* serotypes as well as epidemiological investigation of outbreaks ^12– 14^. In the present study, we defined the population structure and associated genotypic features of *Salmonella* ser. Mbandaka in a global context using whole genome sequence (WGS)-based analysis of 403 *Salmonella* ser. Mbandaka genomes. We assessed the antimicrobial resistance (AMR) and virulence gene repertoire of this *Salmonella* serotype to understand the potential capability of this serovar to act as an important zoonotic pathogen and public health hazard. To verify the genotypic prediction of the AMR and virulence, we examined these characteristics phenotypically using an available set of isolates that represented the study population of 403 *Salmonella* ser. Mbandaka isolates.

## Results

### WGS-based analysis identifies ST413 as the most common *Salmonella* ser. Mbandaka sequence type

 To define the phylogenomic characteristics of *Salmonella* ser. Mbandaka, we used genome sequence data previously deposited in the National Center for Biotechnology Information (NCBI) sequence read archive (SRA) in conjunction with our newly sequenced genomes. To ascertain their serotype as *Salmonella* ser. Mbandaka with an antigenic formula of z10 e,n,z15, we performed *in silico* genoserotyping of all genomes using the web-accessible tool *Salmonella In Silico* Typing Resource (SISTR) ^15^. Samples that showed a discrepancy in their serotype between the available metadata and our serotyping results were removed from further study. This resulted in a final set of 403 *Salmonella* ser. Mbandaka genomes, of which 66 were newly sequenced and the remaining 337 were accessed from the NCBI-SRA. Based on their isolation sources, we grouped them into 12 different categories ( Table 1; Supplementary Table 1, *Underlying data* ^16^).

**Table 1.** Summary of the metadata and genome assembly statistics for the 403 *Salmonella* ser. Mbandaka isolates used in the present study. The distribution of selected isolates based on their geographical origin and isolation source.

Metadata summary			Genome assembly summary		
Origin	Isolation source	Number of genomes	Genome assembly length (Mb) *	Contig number *	N50 (Kb) *
Asia	Animal Feed	2	4.8	109.5	370.35
	Fish	2	4.7	70.5	450.83
	Food	28	4.8	89	329.45
	Human	1	4.8	99	197.14
	Poultry	1	4.7	57	447.16
Africa	Poultry	1	4.8	117	160.28
Europe	Environmental	2	4.7	99	152.32
	Food	9	4.7	102	150.88
	Human	52	4.8	108	167.38
	Poultry	1	4.7	127	150.88
North America	Animal Feed	23	4.8	92.5	324.52
	Bovine	74	4.9	107	235.86
	Canine	6	4.7	131	322.31
	Environmental	24	4.8	87	290.53
	Equine	3	4.8	84	446.56
	Food	54	4.9	102	322.31
	Human	3	4.8	81	260.51
	Porcine	26	4.8	94	222.60
	Poultry	78	4.8	92	265.93
	Wild bird	2	4.7	70.5	353.33
	Other	5	4.7	92	322.60
South America	Animal Feed	1	4.7	91	447.23
	Environmental	2	4.8	154	285.84
	Food	1	4.8	114	322.60
Unknown	Poultry	1	4.7	85	445.94
	Other	1	4.8	110	168.99

*Median

We performed core genome multilocus sequence typing (cgMLST) analysis of the genomes using the web tool ‘SISTR’ and identified five different sequence types (STs) in *Salmonella* ser. Mbandaka. ST413 was the most common type (93% of the isolates) followed by ST1602 (5% of the isolates) (Supplementary Table 2, *Underlying data* ^16^). ST413 showed a global prevalence, while others were limited to certain geographical areas. For example, ST1602, ST2238, and ST2444, were found only in European or Asian isolates, while ST2404 was found only in North American isolates (Supplementary Figure 1, *Extended data* ^17^). A similar trend was observed in the case of isolation sources of these STs. ST143 showed a wide distribution of isolation sources, while a narrower source distribution was found in other STs of this serotype (Supplementary Figure 1, *Extended data* ^17^).

### Phylogenomic analysis of *Salmonella* ser. Mbandaka shows biphasic clustering in its population structure

**Figure 1.**
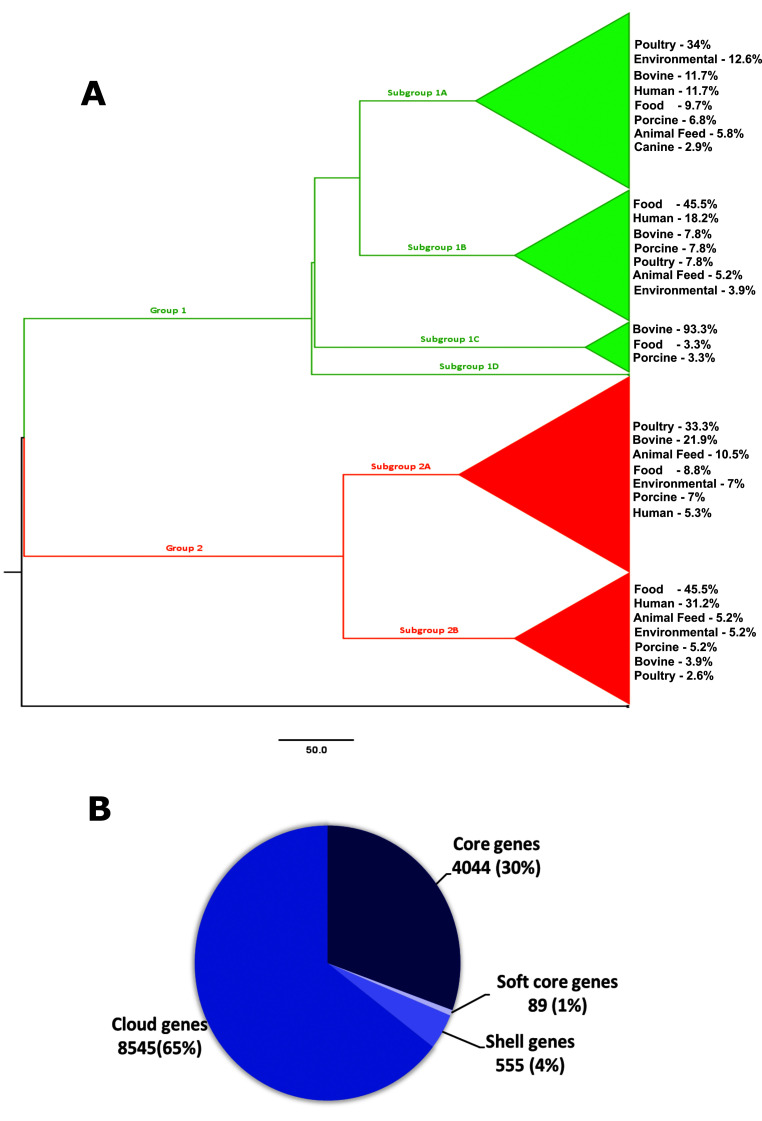
Population structure and pan-genome of *Salmonella* ser. Mbandaka. A. Monomorphic single nucleotide polymorphism (SNP) sites extracted from the multi-FASTA alignment of all the core genes were analyzed for the reconstruction of *Salmonella* ser. Mbandaka phylogeny using the Bayesian approach (BEAST. v.2.5.1). Maximum clade credibility (MCC) tree rooted to outgroup isolates (KY1 and ALT1) showed a separation of the total population into two major groups (colored red and green). Figure was generated using FigTree v.1.4.4. **B.**Pan-genome analysis of *Salmonella* ser. Mbandaka isolates using Roary identified almost 4100 genes (29%) as core genes (present in ≥ 99% of the strains).

**Figure 2.**
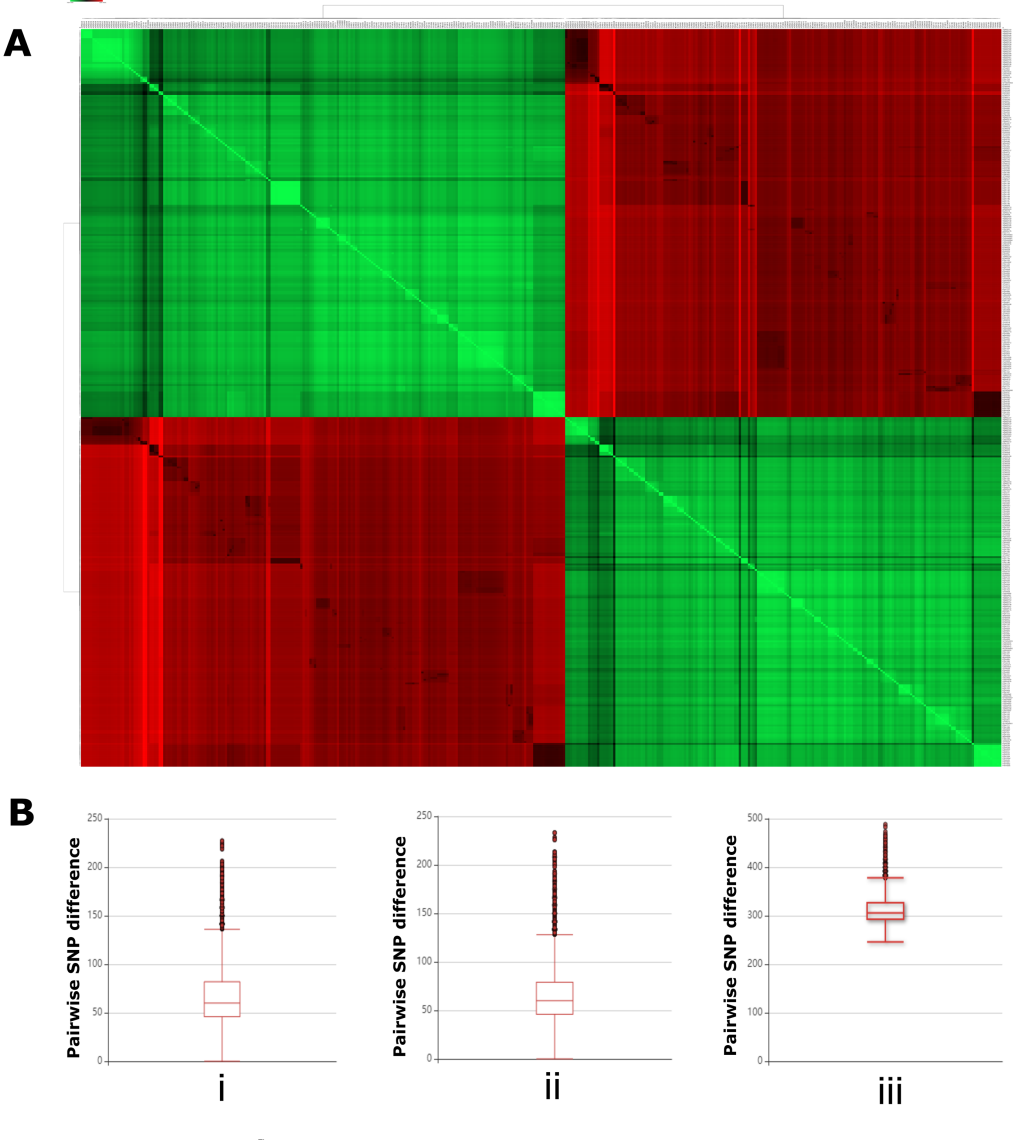
Pairwise single nucleotide polymorphism (SNP) distance between *Salmonella* ser. Mbandaka genomes. **A**) Heatmap showing the hierarchical clustering of 403 *Salmonella* ser. Mbandaka genomes based on the Pearson correlation of their pairwise SNP distance. **B)** Box plot representation of the number of pairwise SNP differences between members of group 1 ( **i**), group 2 ( **ii**), and group 1 and group 2 ( **iii**).

We hypothesized that the population structure of *Salmonella* ser. Mbandaka may have host-specific clades. To test our hypothesis, we constructed a core genome phylogeny and elucidated the pan-genome of the serotype. Figure 1 illustrates the phylogeny of the serotype as predicted by Bayesian evolutionary analysis sampling trees (BEAST). The results show that the isolates bifurcate into two major groups ( Figure 1A). Contrary to our hypothesis, no major host-specific clades were identified in this analysis. The constituent genomes of groups 1 and 2 did not show any clear pattern with respect to their isolation source, geographical origin, or date of isolation (Supplementary Figure 1, *Extended data* ^17^). We generated a pairwise single nucleotide polymorphism (SNP) distance matrix from the core gene alignment of 403 genomes. Interestingly, hierarchal clustering of these genomes based on the Pearson correlation showed the same biphasic clustering of the genomes ( Figure 2), further supporting the core genome phylogeny. The pairwise SNP difference between the members within a group (either group 1 or group 2) differed by a median of 46 SNPs in both groups. However, the difference was large between group 1 and group 2 members, with a median of 293 SNPs ( Figure 2).

Based on the clustering pattern in the Markov chain Monte Carlo (MCMC) tree, we divided each group into different subgroups and determined the distribution of isolates according to their origin and isolation source. We found that all subgroups contained isolates from multiple sources ( Figure 1A); however, there was a closer association of food isolates with Asian countries and human isolates with Europe. These associations were found in both groups 1 and 2. Pan-genome analysis of *Salmonella* ser. Mbandaka revealed a core genome size of 4,044 genes and a pan-genome size of ~ 13,000 genes. The core genes that represented 30% of the pan-genome were found in ≥ 99% of the genomes analyzed; however, the cloud genes i.e., those present in only < 15% of the total genomes analyzed, represented the major share (65%) of the *Salmonella* ser. Mbandaka pan-genome ( Figure 1B).

###  Genotypic and phenotypic screening for antimicrobial resistance (AMR) genes

The presence of antimicrobial-resistant pathogenic bacteria in food has been addressed as a direct hazard to public health ^18^. We determined the AMR profile of *Salmonella* ser. Mbandaka at both the phenotypic and genomic levels.

**Figure 3.**
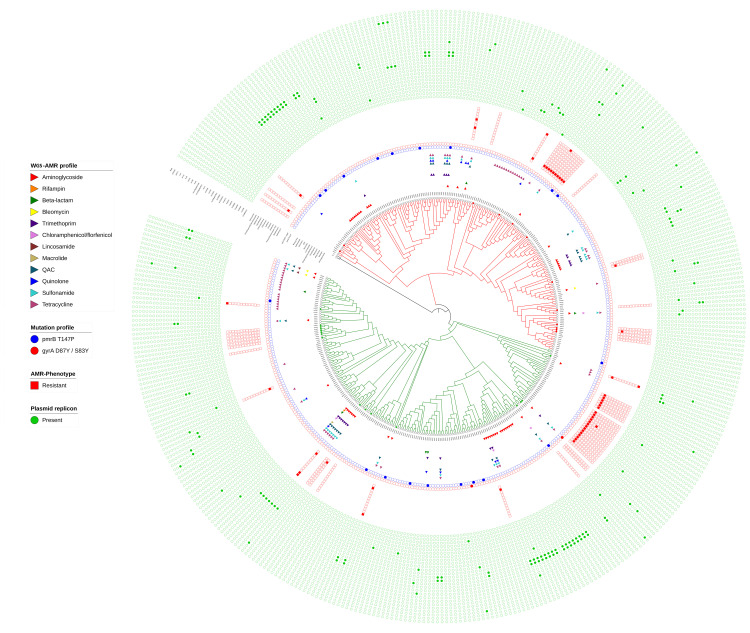
Genotypic and phenotypic prediction of antimicrobial resistance (AMR) in *Salmonella* ser. Mbandaka. AMR genes were predicted from the genomic assemblies and categorized into different groups based on the antibiotic class, as shown in the legend. A cladogram of the MCC tree rooted to outgroup (KY1 and ALT1) is shown at the center. Tree branches are colored to visualize the two major groups. The first circular layer immediately around the tree shows the presence (colored triangle) or absence (no color) of genes resistant to the corresponding antibiotic class. The next two circular layers represent the types of point mutations (pmrB T147B – blue, gyrA D87Y/S83Y – red) identified in *Salmonella* ser. Mbandaka genomes. Newly sequenced isolates (n = 66) formed a representative dataset in the context of their phylogenetic location. The phenotypic resistance profiles of these representative isolates against 12 different antibiotics are depicted in the next circular layer. This is followed by an outermost circular layer that shows the presence (dark) and absence (light) matrix of 26 plasmid replicons in the analyzed genome assemblies. The isolates that showed a match to both phenotypic and genotypic prediction of AMR are marked with a dark color for the tree leaf node. Figure was generated using iTOL v.4.3.2 ^19^.

Our analysis revealed 40 AMR genes in 403 *Salmonella* ser. Mbandaka genomes ( Figure 3; Supplementary Figure 2, *Extended data* ^17^; Supplementary Table 3, *Underlying data* ^16^). These genes were related to resistance against 12 classes of antibiotics. Most resistance was found against tetracycline (16.87% genomes), followed by aminoglycosides (13.89%), sulfonamide (8.4%), QAC (6.9%), trimethoprim (5.7%), and quinolone (3.47%). The gene *tet(B)* (Supplementary Figure 3, *Extended data* ^17^), which confers resistance to the tetracycline group of antibiotics, was most abundant and found in 10.66% of the genomes. This was followed by two aminoglycoside resistance genes *aph(6)-Id* and *aph(3'')-Ib*, which had a percentage occurrence of 8.93% and 8.68%, respectively. We identified isolates carrying resistance genes against quinolones, lincosamide, bleomycin, and rifampin. Thirty-six genomes (8.9%) were found to carry genes conferring resistance against ≥ 3 classes of antimicrobial agents. A total of five quinolone resistance genes ( *qnrB1*, *qnrB19*, *qnrB6*, *qnrB9*, and *qnrS1*) were predicted in 14 isolates. Of the 403 isolates, only six (1.5%) carried genes conferring resistance to β-lactam antimicrobials. The *blaCMY-2*, *blaTEM-1*, and *blaLAP-2* genes were found in three, two, and one genome(s), respectively. These isolates were distributed in the bovine, porcine, food, and human categories of sources and were from North America, Europe and Asia.

We used a representative set of 66 isolates to perform a phenotypic level antibiotic sensitivity assay using a panel of 12 antibiotics [Sensititre™ Gram negative plate (CMV3AGNF, ThermoFisher)]. More than 60% of the isolates showed resistance to at least one antibiotic ( Figure 3). Most resistance was observed against streptomycin (41 isolates, 62%), followed by tetracycline (six isolates, 9%). There were isolates that showed resistance (intermediate) against cefoxitin, chloramphenicol, and sulfonamide; however, no resistance was found against quinolones in these 66 tested isolates (Supplementary Table 4, *Underlying data* ^16^).

Comparison of genotypic predictions with phenotypic susceptibility results found 100% sensitivity for genotypic prediction of phenotypic resistance to nine of 12 antimicrobials, with a specificity of ≥ 95% for all antimicrobials tested ( Table 2). Disagreement was found in 49 (6.2%) of a possible 792 resistance/susceptibility combinations of 12 antimicrobials. The overall specificity was 99%, with that for streptomycin being 100%.

**Table 2.** Comparison of whole genome sequencing-based genotype prediction of antimicrobial resistance versus phenotypic assessment to evaluate the sensitivity and specificity of genotype predictions of resistant phenotypes for a representative set of 66 *Salmonella* ser. Mbandaka isolates. Isolates that showed intermediate resistance to any antimicrobials were also considered resistant.

	Phenotype: resistant (n)		Phenotype: susceptible (n)			
Antimicrobial agent	Genotype: Resistant	Genotype: susceptible	Genotype: Resistant	Genotype: susceptible	Sensitivity (%)	Specificity (%)
Gentamicin	0	0	3	63	100	95.5
Streptomycin	3	38	0	25	7	100
Amoxicillin/Clavulanic Acid	0	0	0	66	100	100
Ampicillin	0	0	0	66	100	100
Cefoxitin	0	2	0	64	0	100
Ceftiofur	0	0	0	66	100	100
Ceftriaxone	0	0	0	66	100	100
Chloramphenicol	0	1	0	65	0	100
Ciprofloxacin	0	0	2	64	100	97
Nalidixic Acid	0	0	2	64	100	97
Trimethoprim/Sulfamethoxazole	1	0	1	64	100	98
Tetracycline	6	0	0	60	100	100
Total	10	41	8	733	20	99
Total- Streptomycin	7	3	8	708	70	99

We subjected all 403 genomes of *Salmonella* ser. Mbandaka to screening of plasmid replicons and point mutations that may confer drug resistance. A total of 26 different plasmids were predicted in our analysis ( Figure 3). With the highest abundance, the IncHI2 and IncHI2A plasmids were predicted in 11.16% of the genomes (Supplementary Figure 4, *Extended data* ^17^; Supplementary Table 5, *Underlying data* ^16^). ColpVC (3.9%), IncFIB(K) (3.47%), and IncI1 (2.23%) were the other predominant plasmids found in *Salmonella* ser. Mbandaka. Using the CLC Genomics Workbench v.12 (Qiagen) and the PointFinder database (accessed on December 2018), we checked chromosomal point mutations associated with AMR in the studied isolates. Of the three types of mutations found, pmrB T147P was the major one, being predicted in 4.7% of genomes. The remaining two were gyrA mutations (gyrA D87Y and S83Y), of which one was detected in a food isolate from Taiwan (FDA187) and the other in a poultry isolate from Nigeria (EUR004) (Supplementary Table 6, *Underlying data* ^16^).

### Assessment of virulence in *Salmonella* ser. Mbandaka isolates shows a uniformity in gene repertoire and related phenotypic characteristics

**Figure 4.**
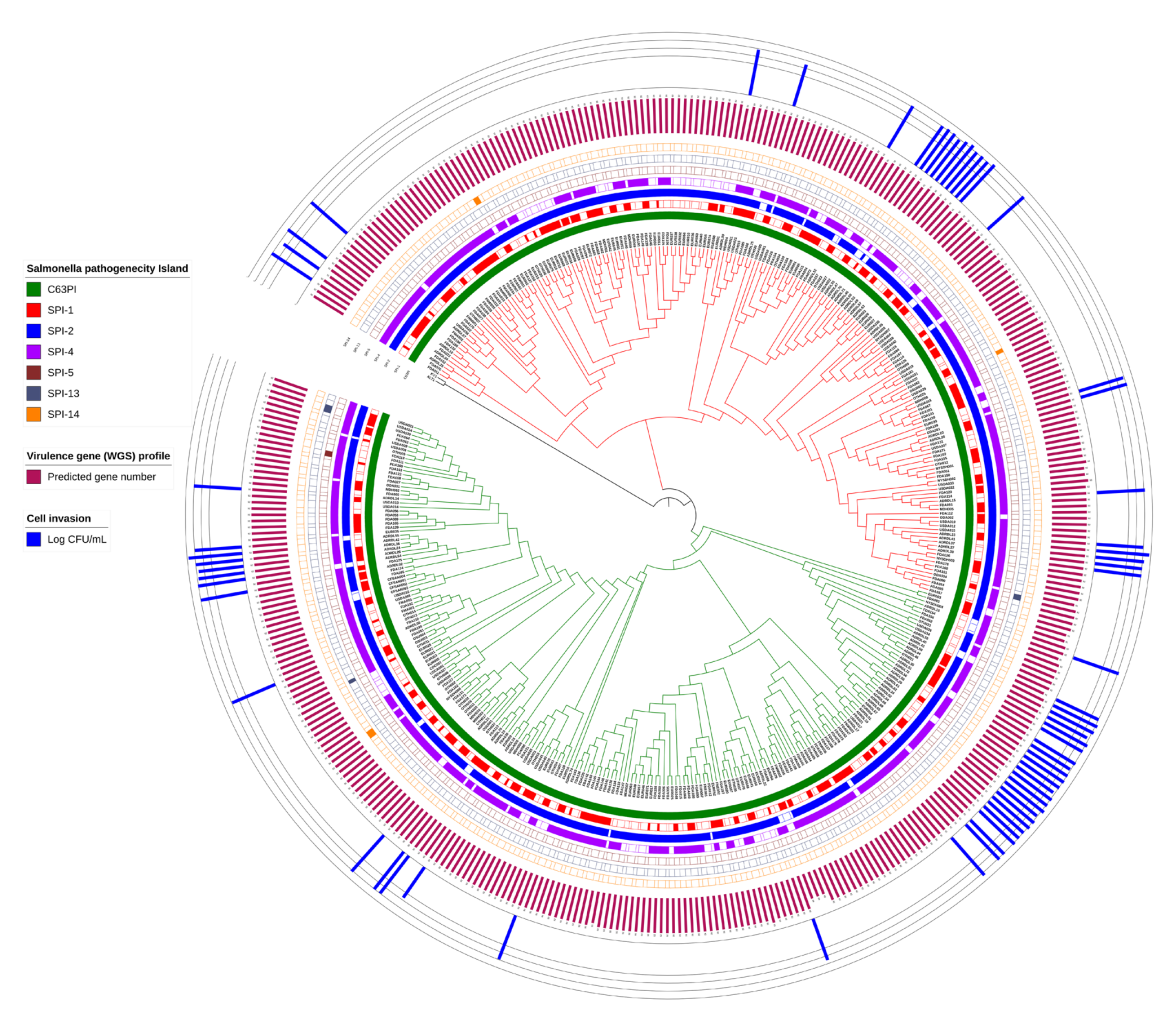
Virulence map of *Salmonella* ser. Mbandaka. Screening of genome assemblies was performed to predict the *Salmonella* pathogenicity islands (SPIs) and virulence factors. A cladogram of the MCC tree rooted to KY1 is shown at the center. Tree branches are colored to visualize the two major groups. The presence (dark color) of different SPIs is shown as different circles around the tree. The number of virulence factors predicted per genome (red) and the number of colony forming units (CFUs) that invaded Caco2 cells (blue) by the representative set of newly sequenced isolates are shown in different circles of simple bar charts. Figure was generated using iTOL v.4.3.2 ^19^.

To determine the potential virulence capability of *Salmonella* ser. Mbandaka isolates, we screened the genomes for the presence of virulence genes and *Salmonella* pathogenicity islands (SPIs). The virulence behavior was further evaluated by an *in vitro* invasion assay in Caco2 cells using a representative set of available isolates from different isolation sources. On average, 92 of the 97 predicted virulence factors were present in all 403 *Salmonella* ser. Mbandaka genomes ( Figure 4; Supplementary Figure 5, *Extended data* ^17^; Supplementary Table 7, *Underlying data* ^16^). With 95% homology, the number of virulence determinants ranged from 89 to 93, with a median value of 92. This indicates that the virulence gene repertoire of the studied genomes was homogenous, irrespective of isolation host or geographical region. A screening of different SPIs was performed using SPIFinder v.1.0 (Center for Genomic Epidemiology). Of the 23 SPIs previously reported in *Salmonella* ^10, 20^, we identified seven (C63PI, SPI-1, SPI-2, SPI-4, SPI-5, SPI-13, and SPI-14) in these genomes ( Figure 4; Supplementary Table 8, *Underlying data* ^16^). Centisome 63 pathogenicity island (C63PI) was found in all 403 genomes. SPI-5 was found only in one poultry isolate (FDA166) collected from the U.S. SPI-13 and SPI-14 were predicted in only a few genomes (each were found in three different isolates) from different sources. We could not identify CS54, SPI-3, SPI-6, SPI-9, SPI-11, SPI-12, or SPI-18 reported in a previous comparative genomic analysis of two *Salmonella* ser. Mbandaka strains ^10^. However, prediction of SPI-13, SPI-14, and C63PI islands in our analysis is in agreement with results obtained from WGS mapping of SPIs in the *Salmonella* ser. Mbandaka ATCC 51958 strain performed in another study ^21^. In accordance with these two previous analyses, we could not predict the presence of SPI-7, 8, 10, 15, 16, 17, 19, 20, 21, or 22 in *Salmonella* ser. Mbandaka genomes.

Host cell invasion and survival in the acidic environment of phagosomes are two critical steps in *Salmonella* pathogenicity; therefore, we used these as surrogate measurements of their phenotypic behavior inside the host. Consistent with our genomic prediction showing uniformity of virulence factors, all tested isolates displayed a similar ability to invade Caco2 cells ( Figure 4; Supplementary Figure 6, *Extended data* ^17^ *;* Supplementary Table 9, *Underlying data* ^16^) and to survive under low pH conditions (Supplementary Figure 7, *Extended data* ^17^ *;* Supplementary Table 10, *Underlying data* ^16^). All 66 tested isolates showed an increase in their growth after three and six hours of exposure to an acidic environment without any prior adaptation. Taken together, the genomic and phenotypic results show the potential virulence capability of *Salmonella* ser. Mbandaka isolates, irrespective of their isolation source, geographical location, and population structure.

## Discussion

In *Salmonella enterica* species, host specificity and the ability to cause disease in different hosts are serotype-dependent ^22^. Some serotypes are “host-restricted,” that is, they are only able to infect one specific host ^23^; others such as *Salmonella* ser. Mbandaka have a broad host range. In addition to humans and farm animals, the main sources of *Salmonella* ser. Mbandaka are dogs, wild birds, and fish. According to outbreak investigation reports, *Salmonella* ser. Mbandaka can originate from both live animals ^8, 24^ and processed food ^7, 9^. Geographically, as well as host distribution wise, ST413 was the most prevalent sequence type in *Salmonella* ser. Mbandaka. Association of ST413 with sources such as animal feed, livestock, food, and humans aids its survival in the food chain and renders *Salmonella* ser. Mbandaka a serious threat for foodborne outbreaks. Unlike other serotypes ^25^, we could not find any specific pattern in the *Salmonella* ser. Mbandaka population structure in relation to either geographical origin or isolation source, disproving our hypothesis that host-specific clades may be emerging in this serotype ( Figure 1A). We presume that this was not due to sampling bias, since a similar clustering pattern was observed in a previous study ^4^ based on pulsed-field gel electrophoresis (PFGE) profiles of a smaller number (n = 70) of *Salmonella* ser. Mbandaka isolates from a geographically restricted area.

Antibiotic use in agricultural settings and animal-based food production provides major contributions to the overall problem of antibiotic resistance ^26^. Due to the widespread use of antimicrobial agents in livestock farming, resistant *Salmonella* strains are more frequently found in animals used for food ^22, 27^. WGS is an excellent technique for the prediction of AMR due to its fast turnaround and affordability. It has been proven to be a successful method for genotypic AMR prediction in several gastrointestinal pathogens including *Salmonella* ^28– 31^. Our study also shows a high sensitivity and specificity for the comparison of genotypic prediction using WGS with phenotypic resistance to nine antimicrobials out of the 12 tested. There was a high discrepancy in the case of streptomycin resistance, since we found 38 isolates that were phenotypically resistant but genotypically susceptible. There could be two reasons for this. Firstly, we used a low minimum inhibitory concentration (MIC) breakpoint of ≥ 16, since there is not a precise clinical breakpoint for streptomycin susceptibility in *Salmonella* ^32^. Secondly, there may exist unknown resistance mechanisms or resistance determinants, that may be absent in the reference database used for prediction ^28^.

Plasmids are one of the main vehicles for dissemination of AMR genes. Resistance genes are assembled on plasmids as arrays by transposition and site-specific recombination mechanisms ^33^. For example, the AMR genes *blaTEM*, *tetA*, *tetB*, and *tetC* have been found to be associated with plasmids in *Salmonella* ser. Typhimurium. Acquisition of plasmids is not a universal phenomenon in all *Salmonella enterica* subspecies *enterica* serotypes. There are many serotypes in this subspecies that do not possess any plasmids ^34^. Above all, animals used for food and food products have been reported as major sources of AMR plasmids ^35^. We predicted 26 different plasmids in 403 draft genomes, of which incompatibility group HI2 (IncHI2) plasmids were the most predominantly identified in our analysis ( Figure 3; Supplementary Figure 4, *Extended data* ^17^; Supplementary Table 5, *Underlying data* ^16^). The presence of IncHI2 plasmids in antibiotic-resistant *Salmonella*, as well as their association with MDR in *Salmonella*, has been reported previously ^36, 37^. In addition, we found the presence of chromosomal mutations associated with quinolone resistance ^38, 39^ (gyrA D87Y and S83Y) and resistance to colistin ^40^ (pmrB), an antibiotic that can be used against carbapenemase-producing Enterobacteriaceae as a last-resort treatment option ^41^. This indicates the potential capability of these mobile genetic elements to spread AMR among *Salmonella*.

Prediction of virulence determinants in *Salmonella* ser. Mbandaka in the present analysis revealed similar virulence gene distribution in all 403 genomes ( Figure 4; Supplementary Figure 5, *Extended data* ^17^; Supplementary Table 7, *Underlying data* ^16^). In addition to virulence factors, we also made use of WGS-based genotypic prediction to elucidate the distribution of pathogenicity islands, large distinct regions on chromosomes that contain virulence genes ^42^. Of the 23 previously reported SPIs in *Salmonella* ^10, 20^, seven were detected in our study isolates, including C63PI ( Figure 4; Supplementary Table 8, *Underlying data* ^16^). *Salmonella* virulence factors are necessary for *Salmonella* pathogenicity, which involves survival in the extreme acidic environment of the host’s stomach, host cell invasion, and survival inside the acidic vacuoles of host immune cells (macrophages) ^42, 43^. Since the results of the cell invasion assay using a representative dataset of available isolates showed similar invasiveness, the virulence at the genomic and phenotypic levels shows high correlation. This may indicate that any *Salmonella* ser. Mbandaka strains may have the potential to cause human infection if ingested by a susceptible individual.

## Methods

### Whole genome sequencing and data acquisition

To study the phylogeny of *Salmonella* ser. Mbandaka, we sequenced 66 isolates (sample name ADRDL-01 to ADRDL-76 in Supplementary Table 1, *Underlying data* ^16^) of this serotype collected from various centers. Genomic DNA was extracted from the overnight cultures of these isolates using a Qiagen DNeasy blood and tissue kit (Qiagen, Inc., Valencia, CA; Cat no. 69506) according to the manufacturer’s protocol and stored at -20°C until use. For WGS, all 66 DNA samples were processed using a Nextera XT DNA sample preparation kit (Illumina inc. San Diego, CA; Cat no. FC-131-1096). Following bead-based normalization, DNA libraries were pooled at an equal volume and sequenced with Miseq reagent (version 2.0) (Illumina Inc., CA) on an Illumina Miseq platform using 2× 250 bp paired-end V2 chemistry. Genome sequences of the remaining isolates (n = 337) were downloaded from the NCBI-SRA (National Center for Biotechnology Information – Sequence Read Archive) database using the sra tool kit v.2.8.1-2. Prior to further analysis, we verified the serotype (Supplementary Table 2, *Underlying data* ^16^) and assembly statistics ( Table 1) of the 403 selected *Salmonella* ser. Mbandaka genomic data using *in silico* methods- *Salmonella* In Silico Typing Resource ( SISTR) ^15^ and assembly-stats, respectively. Metadata for all 403 isolates used in the present study are shown in Supplementary Table 1 (see *Underlying data* ^16^).

### Genome sequence data analysis

Sequencing reads from the *Salmonella* isolates used in the present study ( *Salmonella* ser. Mbandaka n = 403, *Salmonella* ser. Kentucky n = 1, and *Salmonella* ser. Altona n = 1) were assembled into contigs using SPAdes v.3.0 ^44^. To ensure that the assemblies were not greatly fragmented, those containing more than 500 contigs (minimum contig length: 200 bp) were removed from the analysis. *In silico* serotyping and multilocus sequence typing (MLST) of all genomes were performed using the SISTR webserver ^15^. Annotation of genomes was performed using Prokka v1.12 ^45^. A genus-specific database for *Salmonella* was created using a manually annotated reference genome assembly of *Salmonella enterica* ser. Typhimurium str. LT2 (RefSeq assembly accession: GCF_000006945.2) and formatted to a Prokka database as described elsewhere ( https://github.com/tseemann/prokka). Prokaryote pan-genome analysis pipeline Roary v.3.12.0 ^46^ was used for pan-genome analysis and the generation of a multi-FASTA alignment of core genes from the isolates using the aligner PRANK ^47^. The software SNP-sites v2.4.0 ^48^ was used to format the core gene alignment output from Roary to remove gaps and N characters (suitable format for BEAST2 phylogeny). A pairwise SNP distance matrix was created using snp-dists v0.6.3.

To infer the phylogenetic relationship of *Salmonella* ser. Mbandaka isolates, we used the Bayesian maximum clade credibility approach. For this purpose, we used the BEAST2 (v.2.5.1) platform, which employs the MCMC ^49, 50^ method for phylogenetic tree inference. We performed a model testing of the alignment of all SNPs using ‘ ModelTest-NG.v.0.1.5’ and generated a maximum likelihood tree using a generalized time-reversible (GTR+I+G4) substitution model. This tree was constructed to infer the relationship between genetic divergence and time using Tempest ^51^. The resulting phylogeny provided a weak temporal signal (R < 0.10); therefore, tip dates were not included in the final BEAST2 phylogeny. Multiple analyses using both relaxed clock (Log normal) and strict clock models were carried out in combination with coalescent constant population for priors. A MCMC chain length of 100 million generations with 10% preburnin and sampling at every 1000 generations were used for each analysis. The traces from each analysis were examined using Tracer v.1.7.1 ^52^ and the strict clock coalescent constant population model, which showed a better convergence and > 100 effective sample sizes (ESSs) for many of the traces, was selected as a best-fit model for our dataset. Information from 100,000 sample trees produced by BEAST2, after removing 10% burnin, was summarized to a final target MCC tree using TreeAnnotator v2.5.1. We used two other *Salmonella* serotypes [ *Salmonella* ser. Kentucky (KY1) and *Salmonella* ser. Altona (ALT1)] as outgroups, since said serotypes were identified as the nearest neighbors to *Salmonella* ser. Mbandaka by SISTR ^15^.

Antimicrobial resistance (AMR) and virulence gene homologs were identified in assemblies using ABRicate. A minimum sequence identity of 95% and a coverage of 60% were used against the NCBI Bacterial Antimicrobial Resistance Reference Gene Database and Virulence Factor Database (VFDB) for AMR gene prediction and virulence gene profiling, respectively. PlasmidFinder v.2.0 (Center for Genomic Epidemiology) and SPIFinder v.1.0 (Center for Genomic Epidemiology) were used for screening plasmid replicons and Salmonella pathogenicity islands (SPIs) in the genome assemblies ^53^. Point mutations were identified using the CLC Genomics Workbench v.12 (Qiagen) after downloading the PointFinder database for *Salmonella* (accessed on December 2018) *.* An open source alternative for finding point mutation is ResFinder 4.0 offered by the Center for Genomic Epidemiology.

### Antibiotic sensitivity assay

Susceptibility to 12 antimicrobial agents was determined for 66 *Salmonella* ser. Mbandaka isolates using Sensititre™ Gram negative plates (CMV3AGNF, ThermoFisher). Resistance to antimicrobial agents was determined in accordance with the Clinical and Laboratory Standards Institute (CLSI) and National Antimicrobial Resistance Monitoring System (NARMS) guidelines. Five beta lactams (amoxicillin/clavulanic acid, ampicillin, cefoxitin, ceftiofur, and ceftriaxone), two quinolones (ciprofloxacin and nalidixic acid), two aminoglycosides (gentamicin and streptomycin), trimethoprim/sulfamethoxazole, and chloramphenicol were the antibiotics included in the screening panel. Isolates reported as displaying intermediate resistance to any antimicrobials were also considered as resistant.

### Caco2 cell culture and invasion assay

Human colorectal adenocarcinoma (Caco2) cells obtained from ATCC were used for the cell invasion assay. Cells were seeded onto a 24-well plate at a density of 0.3 × 10 ^5^/well and were grown in DMEM medium containing glutamine (DMEM (1×) + glutamine; Gibco) supplemented with 10% (v/v) FBS and 1% antibiotic and antimycotic solution at 37°C with 5% CO _2_ (v/v) for 48 hours. Overnight cultures of bacteria were used to infect Caco2 cell monolayers at a multiplicity of infection (MOI) of 1:100. The invasion assay described by Lee *et al.* ^54^ was used with some modifications. Briefly, bacteria were resuspended in cell culture medium (DMEM (1×) + glutamine; Gibco, with no supplementation) after washing with sterile PBS. Cells were subsequently incubated with this bacterial suspension for 2 hours at 37°C with 5% CO _2_. After infection, the media was removed, and the cells were washed with sterile PBS. Cells were then treated with 400 µL DMEM supplemented with the antibiotic gentamicin (100 µg/mL) to kill extracellular non-invading bacteria. Plates were incubated for 1 hour at 37°C with 5% CO _2_ followed by washing with sterile PBS. A colony forming unit (CFU) count of intracellular bacteria was taken using serial dilution and plating. Cells were lysed with 1% Triton X-100 (Sigma) for 10 minutes to release intracellular bacteria, following which the cell lysate was serially diluted, plated on LB plates, and incubated overnight. Two independent assays, each in triplicate, were performed for each *Salmonella* ser. Mbandaka isolate (total n = 66).

### pH sensitivity assay

An overnight bacterial culture with an OD _600_ adjusted to 0.4 was used to inoculate (20% v/v) low pH LB broth (pH = 4.0 ± 0.1, adjusted using 1M HCl). The experiment was performed in flat- bottomed, non-treated 96-well plates, in which triplicate wells were used for each bacterial sample. The OD _600_ was measured using an ELISA plate reader (BioTek, ELx808) to assess the growth of bacteria over time. The initial OD _600_ was taken immediately after inoculation (T _0_) and then after three hours (T _1_) and six hours (T _2_) of incubation at 37°C in an aerobic environment. The assay was performed as two independent experiments for all 66 isolates.

## Data availability

### Underlying data

The raw sequence data for *Salmonella* strains sequenced in this study have been deposited in NCBI sequence read archive (NCBI SRA) for public access. The full list of NCBI SRA accession numbers is given Supplementary Table 1 (see below).

Zenodo: Underlying Data: Population structure of *Salmonella* serotype Mbandaka. https://doi.org/10.5281/zenodo.4004970 ^16^.

This project contains the following underlying data:

- **Supplementary Table 1.xlsx. Metadata for the 403 *Salmonella* ser. Mbandaka isolates used in the present study.** Major information on the isolates, including both the biosample and SRA run number. With the exception of newly sequenced isolates (n = 66), sequence data for all other isolates in the FASTQ format was acquired with the help of NCBI SRA toolkit.v.2.8.1-2. using the SRA run number. More detailed metadata for individual isolates can be obtained from the NCBI database using the biosample number. (XLSX format)
- **Supplementary Table 2.xlsx. *In silico* serotyping and sequence typing of the 403 *Salmonella* ser. Mbandaka genomes using SISTR.** Detailed results of WGS-based serotype and sequence type prediction of the 403 *Salmonella* ser. Mbandaka genomes using SISTR. (XLSX format)
- **Supplementary Table 3.xlsx. Details of antimicrobial resistance genes predicted in *Salmonella* ser. Mbandaka genomes.** Details of genes identified in the genomes following a search of the NCBI Bacterial Antimicrobial Resistance Reference Gene Database using ABRicate. A gene was reported as present if there were 60% coverage and 95% homology. (XLSX format)
- **Supplementary Table 4.xlsx.** Phenotypic assay results of antimicrobial susceptibility using Sensititre™ Gram negative MIC plates. (XLSX format)
- **Supplementary Table 5.xlsx.** Details of plasmids identified in the *Salmonella* ser. Mbandaka genomes following a search using the PlasmidFinder-2.0 web tool. A sequence identity of 95% and a minimum coverage of 60% were the criteria used for a positive hit. (XLSX format)
- **Supplementary Table 6.xlsx.** Details of the point mutations identified in the *Salmonella* ser. Mbandaka genomes using CLC genomics workbench v.12 (Qiagen) after downloading the PointFinder database for *Salmonella.* (XLSX format)
- **Supplementary Table 7.xlsx.** Details of virulence determinants identified in the genomes following a search of the virulence factor database using ABRicate. A gene was reported as present if there were 60% coverage and 95% homology. (XLSX format)
- **Supplementary Table 8.xlsx.** Details of *Salmonella* pathogenicity island (SPI) genes identified in the genomes following a search using SPIFinder.v.1.0. A gene was reported as present if there were 60% coverage and 95% homology. (XLSX format)
- **Supplementary Table 9.xlsx.** Bacterial count (CFU/mL) of *Salmonella* ser. Mbandaka isolates that invaded Caco2 cells in *in-vitro* cell invasion assay. (XLSX format)
- **Supplementary Table 10.xlsx.** Growth of *Salmonella* ser. Mbandaka in low pH condition was assessed by OD _600_ readings taken at three different time points after inoculation. (XLSX format)

### Extended data

 Zenodo: Extended Data: Population structure of *Salmonella* serotype Mbandaka. https://doi.org/10.5281/zenodo.4005008 ^17^.

This project contains the following extended data:

- **Supplementary Figure 1. Defining the population structure of *Salmonella* ser. Mbandaka isolates.**

An MCC tree of 403 *Salmonella* ser. Mbandaka isolates along with two outgroup strains of *Salmonella* ser. Kentucky (KY1) and *Salmonella* ser. Altona (ALT1). The tree was rerooted to outgroup strains as shown in the circular cladogram. The tree is colored based on the two major groups identified in the phylogeny ( Figure 1). Different circles around the tree represent cgMLST sequence type as well as different metadata attributes of the genomes. Figure was generated using iTOL v.4.3.2 ^19^. (PNG format)

- **Supplementary Figure 2. Antimicrobial resistance gene prediction in *Salmonella* ser. Mbandaka isolates.**

Heat map showing the predicted AMR genes (n = 40) in the *Salmonella* ser. Mbandaka isolates. The dark color represents the presence of a predicted gene at 90% sequence identity with a minimum coverage of 60%. The tree represents the MCC tree created using BEAST v.2.5.1. Figure was generated using iTOL v.4.3.2 ^19^. (PNG format)

- **Supplementary Figure 3. Abundance distribution of AMR genes in the 403 *Salmonella* ser. Mbandaka isolates.**

Simple bar graph showing the percentage of *Salmonella* ser. Mbandaka genomes harboring each predicted AMR gene. More than 10% of genomes carried the tet(B) gene that confers resistance to tetracycline antibiotics, followed by the aminoglycoside resistance genes aph(6)-ld and aph(3”)-lb (8.9% and 8.7%, respectively). (PNG format)

- **Supplementary Figure 4. Distribution of plasmid replicons in *Salmonella* ser. Mbandaka.**

IncHI2 and IncHI2A plasmids were found to be the most abundant replicons in *Salmonella* ser. Mbandaka genomes. Simple bar graph with plasmids on the ‘X’ axis and the percentage of genomes on the ‘Y’ axis. (PNG format)

- **Supplementary Figure 5. WGS-based profiling of virulence genes in *Salmonella* ser. Mbandaka.**

Heat map showing the predicted virulence factors in *Salmonella* ser. Mbandaka genomes at a minimum sequence identity of 95% and a minimum coverage of 60%. Virulence factors were categorized into six groups as shown in the color legend. Approximately 87% of predicted genes were found in all 403 *Salmonella* ser. Mbandaka genomes. Figure was generated using iTOL v.4.3.2 ^19^. TTSS (SPI-1) - Type three secretion system encoded by *Salmonella* pathogenicity island-1; TTSS (SPI-2) - Type three secretion system encoded by *Salmonella* pathogenicity island-2. (PNG format)

- **Supplementary Figure 6. Invasiveness of *Salmonella* ser. Mbandaka isolates in Caco2 cells.**

The 66 newly sequenced *Salmonella* ser. Mbandaka isolates were used for the invasion assay in Caco2 cells. Bar plot showing the count of intracellular bacteria (log CFU/mL) retrieved after an incubation time of two hours under aerobic conditions followed by treatment with gentamicin to kill all extracellular bacteria. Cell lysates were serially diluted and plated on LB plates in duplicate. Figure was generated using Prism 7 (GraphPad software, Inc.). (TIF format)

- **Supplementary Figure 7. Adaptation of *Salmonella* ser. Mbandaka isolates to low pH.**

The ability of *Salmonella* ser. Mbandaka isolates to tolerate an acidic environment was tested using the 66 newly sequenced isolates as representatives. All tested isolates were able to withstand the immediate exposure to a low pH environment and showed an increase in growth after three hours (A) and six hours (B) of incubation at 37°C under aerobic conditions. Bar graph showing the OD _600_ immediately after exposure to LB broth at pH 4.0 (T0) and after three hours (T1) and six hours (T2) of incubation. Figure was generated using Prism 7 (GraphPad software, Inc.). (PNG format)

Data are available under the terms of the Creative Commons Attribution 4.0 International license (CC-BY 4.0).

## References

[1] Basler C Forshey TM Machesky K: Multistate outbreak of human *Salmonella* infections linked to live poultry from a mail-order hatchery in Ohio--March-September 2013. *MMWR Morb Mortal Wkly Rep.* 2014;63(10):222. 24622287 PMC5779341

[2] Scallan E Hoekstra RM Angulo FJ: Foodborne illness acquired in the United States--major pathogens. *Emerg Infect Dis.* 2011;17(1):7–15. 10.3201/eid1701.p11101 21192848 PMC3375761

[3] Gragg SE Loneragan GH Brashears MM: Cross-sectional study examining *Salmonella enterica* carriage in subiliac lymph nodes of cull and feedlot cattle at harvest. *Foodborne Pathog Dis.* 2013;10(4):368–74. 10.1089/fpd.2012.1275 23566273 PMC3696922

[4] Hoszowski A Zając M Lalak A: Fifteen years of successful spread of Salmonella enterica serovar Mbandaka clone ST413 in Poland and its public health consequences. *Ann Agric Environ Med.* 2016;23(2):237–41. 10.5604/12321966.1203883 27294625

[5] Hoszowski A Wasyl D: Typing of *Salmonella enterica* subsp. *enterica* serovar Mbandaka isolates. *Vet Microbiol.* 2001;80(2):139–48. 10.1016/s0378-1135(00)00382-5 11295334

[6] Scheil W Cameron S Dalton C: A South Australian Salmonella Mbandaka outbreak investigation using a database to select controls. *Aust N Z J Public Health.* 1998;22(5):536–9. 10.1111/j.1467-842x.1998.tb01434.x 9744205

[7] Centers for Disease Control and Prevention (CDC): Multistate Outbreak of *Salmonella* Montevideo and *Salmonella* Mbandaka Infections Linked to Tahini Sesame Paste (Final Update).2013 Reference Source

[8] Centers for Disease Control and Prevention (CDC): Eight Multistate Outbreaks of Human *Salmonella* Infections Linked to Live Poultry in Backyard Flocks (Final Update).2016 Reference Source

[9] Centers for Disease Control and Prevention (CDC): Multistate Outbreak of *Salmonella* Mbandaka Infections Linked to Kellogg’s Honey Smacks Cereal (Final Update).2018 Reference Source

[10] Hayward MR Jansen VAA Woodward MJ: Comparative genomics of *Salmonella enterica* serovars Derby and Mbandaka, two prevalent serovars associated with different livestock species in the UK. *BMC Genomics.* 2013;14:365. 10.1186/1471-2164-14-365 23725633 PMC3680342

[11] Hayward MR Petrovska L Jansen VAA: Population structure and associated phenotypes of *Salmonella enterica* serovars Derby and Mbandaka overlap with host range. *BMC Microbiol.* 2016;16(1):15. 10.1186/s12866-016-0628-4 26846255 PMC4743429

[12] den Bakker HC Switt AIM Cummings CA: A whole-genome single nucleotide polymorphism-based approach to trace and identify outbreaks linked to a common *Salmonella enterica* subsp. *enterica* serovar Montevideo pulsed-field gel electrophoresis type. *Appl Environ Microbiol.* 2011;77(24):8648–55. 10.1128/AEM.06538-11 22003026 PMC3233099

[13] Wilson MR Brown E Keys C: Whole Genome DNA Sequence Analysis of *Salmonella* subspecies *enterica* serotype Tennessee obtained from related peanut butter foodborne outbreaks. *PLoS One.* 2016;11(6):e0146929. 10.1371/journal.pone.0146929 27258142 PMC4892500

[14] Leekitcharoenphon P Nielsen EM Kaas RS: Evaluation of whole genome sequencing for outbreak detection of *Salmonella enterica*. *PLoS One.* 2014;9(2):e87991. 10.1371/journal.pone.0087991 24505344 PMC3913712

[15] Yoshida CE Kruczkiewicz P Laing CR: The *Salmonella In Silico* Typing Resource (SISTR): An Open Web-Accessible Tool for Rapidly Typing and Subtyping Draft *Salmonella* Genome Assemblies. *PLoS One.* 2016;11(1):e0147101. 10.1371/journal.pone.0147101 26800248 PMC4723315

[16] Antony L Fenske G Thomas M: Underlying Data: Population structure of Salmonella serotype Mbandaka [Data set]. *Zenodo.* 2020 10.5281/zenodo.4004970 PMC7653644 33214877

[17] Antony L Fenske G Thomas M: Extended Data: Population structure of Salmonella serotype Mbandaka. *Zenodo.* 2020 10.5281/zenodo.4005008 PMC7653644 33214877

[18] Collineau L Boerlin P Carson CA: Integrating Whole-Genome Sequencing Data Into Quantitative Risk Assessment of Foodborne Antimicrobial Resistance: A Review of Opportunities and Challenges. *Front Microbiol.* 2019;10:1107. 10.3389/fmicb.2019.01107 31231317 PMC6558386

[19] Letunic I Bork P: Interactive tree of life (iTOL) v3: an online tool for the display and annotation of phylogenetic and other trees. *Nucleic Acids Res.* 2016;44(W1):W242–5. 10.1093/nar/gkw290 27095192 PMC4987883

[20] Fookes M Schroeder GN Langridge GC: *Salmonella bongori* provides insights into the evolution of the Salmonellae. *PLoS Pathog.* 2011;7(8):e1002191. 10.1371/journal.ppat.1002191 21876672 PMC3158058

[21] Roer L Hendriksen RS Leekitcharoenphon P: Is the Evolution of *Salmonella enterica* subsp. *enterica* Linked to Restriction-Modification Systems? *mSystems.* 2016;1(3):e00009–16. 10.1128/mSystems.00009-16 27822532 PMC5069764

[22] Andino A Hanning I: *Salmonella enterica*: survival, colonization, and virulence differences among serovars. *ScientificWorldJournal.* 2015;2015:520179. 10.1155/2015/520179 25664339 PMC4310208

[23] Jajere SM: A review of *Salmonella enterica* with particular focus on the pathogenicity and virulence factors, host specificity and antimicrobial resistance including multidrug resistance. *Vet World.* 2019;12(4):504–521. 10.14202/vetworld.2019.504-521 31190705 PMC6515828

[24] Centers for Disease Control and Prevention (CDC): Multistate Outbreak of Human *Salmonella* Infections Linked to Live Poultry (Final Update).2013 Reference Source

[25] Makendi C Page AJ Wren BW: A Phylogenetic and Phenotypic Analysis of *Salmonella enterica* Serovar Weltevreden, an Emerging Agent of Diarrheal Disease in Tropical Regions. *PLoS Negl Trop Dis.* 2016;10(2):e0004446. 10.1371/journal.pntd.0004446 26867150 PMC4750946

[26] Landers TF Cohen B Wittum TE: A review of antibiotic use in food animals: perspective, policy, and potential. *Public Health Rep.* 2012;127(1): 4–22. 10.1177/003335491212700103 22298919 PMC3234384

[27] Su LH Chiu CH Chu C: Antimicrobial resistance in nontyphoid *Salmonella* serotypes: a global challenge. *Clin Infect Dis.* 2004;39(4): 546–51. 10.1086/422726 15356819

[28] Neuert S Nair S Day MR: Prediction of Phenotypic Antimicrobial Resistance Profiles From Whole Genome Sequences of Non-typhoidal *Salmonella enterica*. *Front Microbiol.* 2018;9:592. 10.3389/fmicb.2018.00592 29636749 PMC5880904

[29] Stoesser N Batty EM Eyre DW: Predicting antimicrobial susceptibilities for *Escherichia coli* and *Klebsiella pneumoniae* isolates using whole genomic sequence data. *J Antimicrob Chemother.* 2013;68(10):2234–44. 10.1093/jac/dkt180 23722448 PMC3772739

[30] McDermott PF Tyson GH Kabera C: Whole-Genome Sequencing for Detecting Antimicrobial Resistance in Nontyphoidal *Salmonella*. *Antimicrob Agents Chemother.* 2016;60(9):5515–20. 10.1128/AAC.01030-16 27381390 PMC4997858

[31] Sadouki Z Day MR Doumith M: Comparison of phenotypic and WGS-derived antimicrobial resistance profiles of *Shigella sonnei* isolated from cases of diarrhoeal disease in England and Wales, 2015. *J Antimicrob Chemother.* 2017;72(9):2496–2502. 10.1093/jac/dkx170 28591819

[32] Pornsukarom S van Vliet AHM Thakur S: Whole genome sequencing analysis of multiple *Salmonella* serovars provides insights into phylogenetic relatedness, antimicrobial resistance, and virulence markers across humans, food animals and agriculture environmental sources. *BMC Genomics.* 2018;19(1):801. 10.1186/s12864-018-5137-4 30400810 PMC6218967

[33] Bennett PM: Plasmid encoded antibiotic resistance: acquisition and transfer of antibiotic resistance genes in bacteria. *Br J Pharmacol.* 2008;153 Suppl 1(Suppl 1): S347–57. 10.1038/sj.bjp.0707607 18193080 PMC2268074

[34] Rychlik I Gregorova D Hradecka H: Distribution and function of plasmids in *Salmonella enterica*. *Vet Microbiol.* 2006;112(1):1–10. 10.1016/j.vetmic.2005.10.030 16303262

[35] Madec JY Haenni M: Antimicrobial resistance plasmid reservoir in food and food-producing animals. *Plasmid.* 2018;99:72–81. 10.1016/j.plasmid.2018.09.001 30194944

[36] Glenn LM Lindsey RL Folster JP: Antimicrobial resistance genes in multidrug-resistant *Salmonella enterica* isolated from animals, retail meats, and humans in the United States and Canada. *Microb Drug Resist.* 2013;19(3):175–84. 10.1089/mdr.2012.0177 23350745 PMC4665089

[37] Chen W Fang T Zhou X: IncHI2 Plasmids Are Predominant in Antibiotic-Resistant *Salmonella* Isolates. *Front Microbiol.* 2016;7:1566. 10.3389/fmicb.2016.01566 27746775 PMC5043248

[38] Hopkins KL Davies RH Threlfall EJ: Mechanisms of quinolone resistance in *Escherichia coli* and *Salmonella*: recent developments. *Int J Antimicrob Agents.* 2005;25(5):358–73. 10.1016/j.ijantimicag.2005.02.006 15848289

[39] Zankari E Allesøe R Joensen KG: PointFinder: a novel web tool for WGS-based detection of antimicrobial resistance associated with chromosomal point mutations in bacterial pathogens. *J Antimicrob Chemother.* 2017;72(10):2764–2768. 10.1093/jac/dkx217 29091202 PMC5890747

[40] Sun S Negrea A Rhen M: Genetic analysis of colistin resistance in *Salmonella enterica* serovar Typhimurium. *Antimicrob Agents Chemother.* 2009;53(6):2298–305. 10.1128/AAC.01016-08 19332669 PMC2687247

[41] Liu YY Wang Y Walsh TR: Emergence of plasmid-mediated colistin resistance mechanism MCR-1 in animals and human beings in China: a microbiological and molecular biological study. *Lancet Infect Dis.* 2016;16(2):161–8. 10.1016/S1473-3099(15)00424-7 26603172

[42] Hensel M: Evolution of pathogenicity islands of *Salmonella enterica*. *Int J Med Microbiol.* 2004;294(2–3):95–102. 10.1016/j.ijmm.2004.06.025 15493819

[43] Kenney LJ: The role of acid stress in *Salmonella pathogenesis*. *Curr Opin Microbiol.* 2019;47:45–51. 10.1016/j.mib.2018.11.006 30529007

[44] Bankevich A Nurk S Antipov D: SPAdes: a new genome assembly algorithm and its applications to single-cell sequencing. *J Comput Biol.* 2012;19(5):455–77. 10.1089/cmb.2012.0021 22506599 PMC3342519

[45] Seemann T: Prokka: rapid prokaryotic genome annotation. *Bioinformatics.* 2014;30(14):2068–9. 10.1093/bioinformatics/btu153 24642063

[46] Page AJ Cummins CA Hunt M: Roary: rapid large-scale prokaryote pan genome analysis. *Bioinformatics.* 2015;31(22):3691–3. 10.1093/bioinformatics/btv421 26198102 PMC4817141

[47] Loytynoja A: Phylogeny-aware alignment with PRANK. *Methods Mol Biol.* 2014;1079:155–70. 10.1007/978-1-62703-646-7_10 24170401

[48] Page AJ Taylor B Delaney AJ: *SNP-sites*: rapid efficient extraction of SNPs from multi-FASTA alignments. *Microb Genom.* 2016;2(4):e000056. 10.1099/mgen.0.000056 28348851 PMC5320690

[49] Drummond AJ Nicholls GK Rodrigo AG: Estimating mutation parameters, population history and genealogy simultaneously from temporally spaced sequence data. *Genetics.* 2002;161(3):1307–20. 12136032 10.1093/genetics/161.3.1307 PMC1462188

[50] Bouckaert R Heled J Kühnert D: BEAST 2: a software platform for Bayesian evolutionary analysis. *PLoS Comput Biol.* 2014;10(4):e1003537. 10.1371/journal.pcbi.1003537 24722319 PMC3985171

[51] Rambaut, A Lam TT Carvalho LM: Exploring the temporal structure of heterochronous sequences using TempEst (formerly Path-O-Gen). *Virus Evol.* 2016;2(1):vew007. 10.1093/ve/vew007 27774300 PMC4989882

[52] Rambaut A Drummond AJ Xie D: Posterior Summarization in Bayesian Phylogenetics Using Tracer 1.7. *Syst Biol.* 2018;67(5):901–904. 10.1093/sysbio/syy032 29718447 PMC6101584

[53] Carattoli A García-Fernández A Zankari E: *In silico* detection and typing of plasmids using PlasmidFinder and plasmid multilocus sequence typing. *Antimicrob Agents Chemother.* 2014;58(7):3895–903. 10.1128/AAC.02412-14 24777092 PMC4068535

[54] Lee CA Falkow S: The ability of *Salmonella* to enter mammalian cells is affected by bacterial growth state. *Proc Natl Acad Sci U S A.* 1990;87(11):4304–8. 10.1073/pnas.87.11.4304 2349239 PMC54097

