# Supplementary material for: Population structure of Salmonella enterica serotype Mbandaka reveals similar virulence potential irrespective of source and phylogenomic stratification
Source: F1000Res. 2020 Sep 16;9:1142. [Version 1] doi: 10.12688/f1000research.25540.1 (PMC7653644; doi:10.12688/f1000research.25540.1)
Supplement: Peer review history

# Peer review: Reviewer response for version 1

**DOI:** 10.5256/f1000research.28185.r71448

**Published:** 2020-11-09

**Authors:** Shaankumar Mooyottu

**Affiliations:** 1 Department of Veterinary Pathology, College of Veterinary Medicine, Iowa State University, Ames, IA, USA

The current study defines the population structure and associated genotypic features of *Salmonella* ser. Mbandaka in a global context using whole-genome sequence (WGS)-based analysis of 403 *Salmonella* ser. Mbandaka genomes. The authors assessed the antimicrobial resistance and virulence gene repertoire of this *Salmonella* serotype to understand the potential capability of this serovar to act as an important zoonotic pathogen and public health hazard. The research method, results, and presentation look sound, and no significant shortcomings are identified.

Is the work clearly and accurately presented and does it cite the current literature?

Yes

If applicable, is the statistical analysis and its interpretation appropriate?

Yes

Are all the source data underlying the results available to ensure full reproducibility?

Yes

Is the study design appropriate and is the work technically sound?

Yes

Are the conclusions drawn adequately supported by the results?

Yes

Are sufficient details of methods and analysis provided to allow replication by others?

Yes

Reviewer Expertise:

Pathology and microbiology.

I confirm that I have read this submission and believe that I have an appropriate level of expertise to confirm that it is of an acceptable scientific standard.

# Peer review: Reviewer response for version 1

**DOI:** 10.5256/f1000research.28185.r71450

**Published:** 2020-09-25

**Authors:** Mohamed N. Seleem

**Affiliations:** 1 Virginia Polytechnic Institute and State University, Blacksburg, VA, USA

This manuscript describes the whole genome sequencing-based analysis of population structure, antibiotic resistance, and virulence profile of Salmonella Mbandaka, a serotype that primarily colonizes animals but also causes human infections. Authors have sequenced 66 isolates and compared the data against 337 publicly available genomes of S. Mbandaka. A subset of strains were also tested phenotypically to determine virulence and antibiotic resistance profile. The analysis did not show any host specific clustering. The virulence and antibiotic resistance profile was not host or region specific. CaCo2 cell based virulence assay showed that most strains are invasive, indicating that any strain could possibly cause infection in a susceptible host. Overall, this manuscript is written well. However, a few modifications could improve the clarity of the results. Minor points to consider are:

1. Figure 2 is confusing, and the row and column headers are not readable. I assume that row and column headers are isolates. However, that is not mentioned in the legend. Please correct.
2. What is the significance of groups in Figure 2B? Are these groups related to group 1 and 2 in Figure 1? Are you calculating the SNPs in these groups based on Beast phylogeny? Please include the analysis details in the figure legend.
3. The inner labels in Figure 3 are not readable. The figure overall is faint and hard to understand. The font size of the ring labels needs to be increased to make it readable.
4. The same problem is in Figure 4. This figure is better than Figure 3. However, the inner labels are still hard to read.

Is the work clearly and accurately presented and does it cite the current literature?

Yes

If applicable, is the statistical analysis and its interpretation appropriate?

Yes

Are all the source data underlying the results available to ensure full reproducibility?

Yes

Is the study design appropriate and is the work technically sound?

Yes

Are the conclusions drawn adequately supported by the results?

Yes

Are sufficient details of methods and analysis provided to allow replication by others?

Yes

Reviewer Expertise:

Microbiology and antimicrobial resistance.

I confirm that I have read this submission and believe that I have an appropriate level of expertise to confirm that it is of an acceptable scientific standard.
